# Supplementary material for: A Humanized Yeast Phenomic Model of Deoxycytidine Kinase to Predict Genetic Buffering of Nucleoside Analog Cytotoxicity
Source: Genes (Basel). 2019 Sep 30;10(10):770. doi: 10.3390/genes10100770 (PMC6826991; doi:10.3390/genes10100770)
Supplement: Supplementary file 1 [file genes-10-00770-s001.zip › Additional_Files/Additional_File7_GOTermHeatmaps.zip!/Additional_File7_GOTermHeatmaps/protein_tyrosine_phosphatase_activity.pdf]

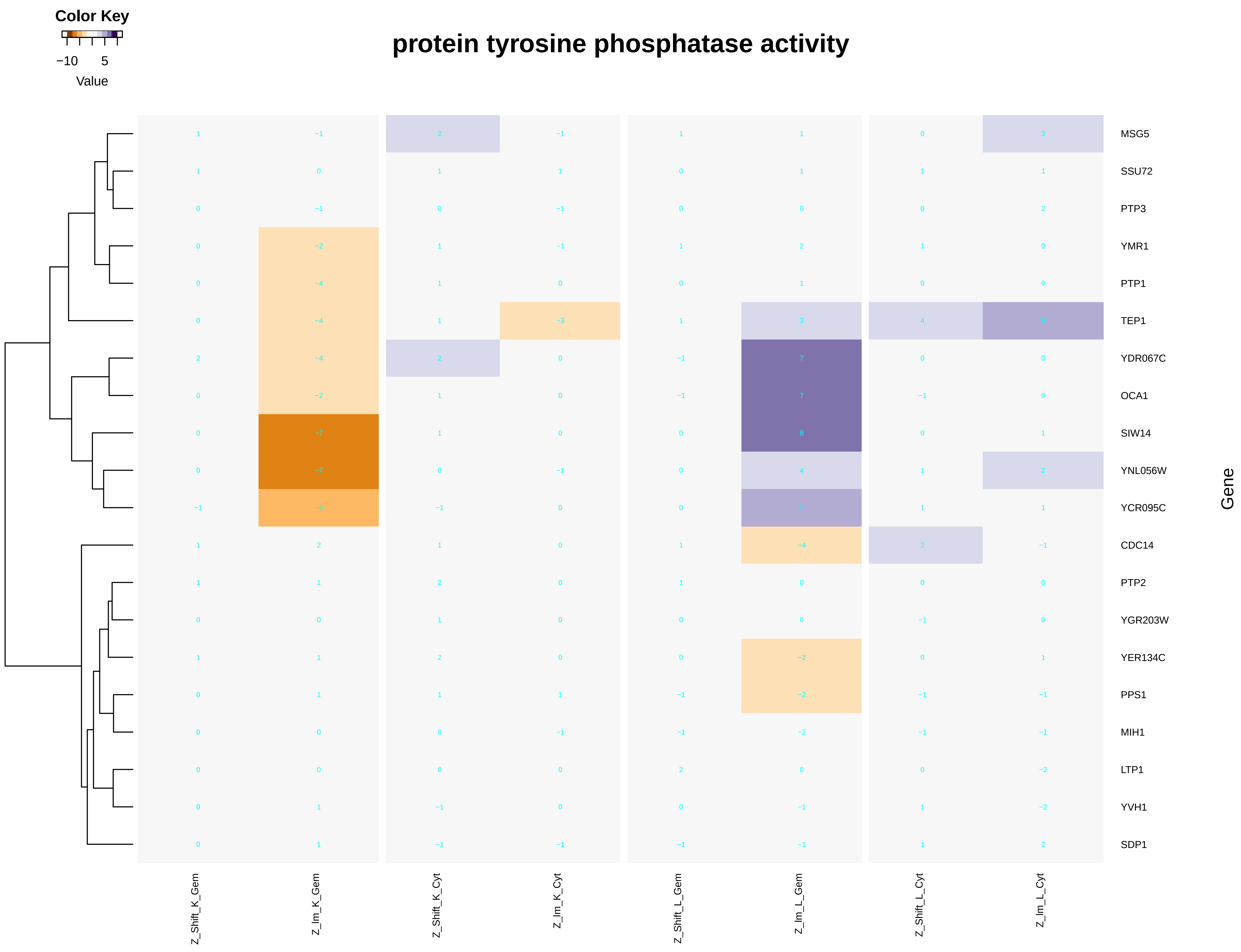
Color Key
−10 5
Value
protein tyrosine phosphatase activity
1 −1 2 −1 1 1 0 3 MSG5
1 0 1 1 0 1 1 1 SSU72
0 −1 0 −1 0 0 0 2 PTP3
0 −2 1 −1 1 2 1 0 YMR1
0 −4 1 0 0 1 0 0 PTP1
0 −4 1 −3 1 3 4 5 TEP1
2 −4 2 0 −1 7 0 0 YDR067C
0 −2 1 0 −1 7 −1 0 OCA1
0 −7 1 0 0 8 0 1 SIW14
0 −7 0 −1 0 4 1 2 YNL056W
−1 −6 −1 0 0 5 1 1 YCR095C
1 2 1 0 1 −4 2 −1 CDC14
1 1 2 0 1 0 0 0 PTP2
0 0 1 0 0 0 −1 0 YGR203W
1 1 2 0 0 −2 0 1 YER134C
0 1 1 1 −1 −2 −1 −1 PPS1
0 0 0 −1 −1 −2 −1 −1 MIH1
0 0 0 0 2 0 0 −2 LTP1
0 1 −1 0 0 −1 1 −2 YVH1
0 1 −1 −1 −1 −1 1 2 SDP1
Gene
Z_Shift_K_Gem
Z_Im_K_Gem
Z_Shift_K_Cyt
Z_Im_K_Cyt
Z_Shift_L_Gem
Z_Im_L_Gem
Z_Shift_L_Cyt
Z_Im_L_Cyt
